# Rod and Cone Connections With Bipolar Cells in the Rabbit Retina

**DOI:** 10.3389/fncel.2021.662329

**Published:** 2021-05-06

**Authors:** Christopher M. Whitaker, Gina Nobles, Munenori Ishibashi, Stephen C. Massey

**Affiliations:** Ruiz Department of Ophthalmology and Visual Science, McGovern Medical School, University of Texas Medical School at Houston, Houston, TX, United States

**Keywords:** retina, rod, cone, AII amacrine cell, bipolar cell

## Abstract

Rod and cone pathways are segregated in the first stage of the retina: cones synapse with both ON- and OFF-cone bipolar cells while rods contact only rod bipolar cells. However, there is an exception to this specific wiring in that rods also contact certain OFF cone bipolar cells, providing a tertiary rod pathway. Recently, it has been proposed that there is even more crossover between rod and cone pathways. Physiological recordings suggested that rod bipolar cells receive input from cones, and ON cone bipolar cells can receive input from rods, in addition to the established pathways. To image their rod and cone contacts, we have dye-filled individual rod bipolar cells in the rabbit retina. We report that approximately half the rod bipolar cells receive one or two cone contacts. Dye-filling AII amacrine cells, combined with subtractive labeling, revealed most of the ON cone bipolar cells to which they were coupled, including the occasional blue cone bipolar cell, identified by its contacts with blue cones. Imaging the AII-coupled ON cone bipolar dendrites in this way showed that they contact cones exclusively. We conclude that there is some limited cone input to rod bipolar cells, but we could find no evidence for rod contacts with ON cone bipolar cells. The tertiary rod OFF pathway operates *via* direct contacts between rods and OFF cone bipolar cells. In contrast, our results do not support the presence of a tertiary rod ON pathway in the rabbit retina.

## Introduction

The mammalian retina can process signals over a vast range of intensities, approximately 10 log units, from starlight to sunlight. In part, this is accomplished by the use of two types of photoreceptors, rods, and cones, which operate in different intensity ranges. Rods are specialized for high sensitivity in dark conditions whereas cones operate in daylight and support color vision.

Rod signals can reach retinal ganglion cells *via* several different pathways (Bloomfield and Völgyi, 2009). In the canonical primary rod pathway, rods signal to rod bipolar cells which then synapse onto AII amacrine cells. In turn, AII amacrine cells split this signal *via* sign-conserving gap junctions with ON cone bipolar cells and inhibitory glycinergic synapses with OFF cone bipolar cells, or sometimes directly with OFF ganglion cells. Finally, the ON and OFF cone bipolar cells relay signals to their respective ON and OFF retinal ganglion cells. This pathway operates as a high gain circuit that facilitates the transmission of single-photon responses from rods. Due to the amplification, it has been reported that rod bipolar cells saturate at relatively low light levels, even before the threshold for cone vision has been reached (Field et al., 2005) In this so-called “mesopic” range of intensities, it is thought that additional circuits are recruited that bypass the rod bipolar cell. For example, in the secondary rod pathway, rod signals pass directly to cones *via* rod/cone gap junctions and this pathway is also active below the cone threshold (Jin et al., 2020). Finally, there is a tertiary rod pathway that bypasses rod bipolar cells by making direct connections between rods and OFF cone bipolar cells (Soucy et al., 1998; Bloomfield and Völgyi, 2009).

However, previous work has challenged this scheme of segregated pathways, providing evidence for more crossover between rod and cone circuits. Physiological recordings from rod bipolar cells suggested that a subset receives direct cone input (Pang et al., 2010). Morphological studies, using confocal microscopy or serial blockface reconstruction, have both confirmed cone contacts with RBCs (Behrens et al., 2016; Pang et al., 2018). In the current study, we have tested the hypothesis that cones contact rod bipolar cells directly in the rabbit retina. Immunohistochemistry revealed putative synapses between cones and rod bipolar cells. To confirm these findings, we dye-injected individual rod bipolar cells and quantified their cone contacts. Our results demonstrated that ~50% of rod bipolar cells receive one or two cone contacts.

Besides the additional cone input to rod pathways, it has also been reported that there is a sustained rod-driven input to cone bipolar cells, including those with responses to light increments (Pang et al., 2010). This implies there may be direct connections between rods and ON cone bipolar cells, in addition to the well-known connections between rods and OFF bipolar cells, which make up the tertiary rod OFF pathway (Tsukamoto et al., 2007). The morphological evidence for rod input to ON cone bipolar cells is mixed with some reports in mouse of rod input to cone bipolar type 7 (Tsukamoto et al., 2007; Keeley and Reese, 2010), while other results were negative in mouse (Haverkamp et al., 2006). In the primate retina, the giant ON bipolar cell also made some rod contacts (Tsukamoto and Omi, 2016).

To address this question in the rabbit retina, we filled the population of ON cone bipolar cells *via* their gap junctions with AII amacrine cells. This method effectively separates ON from OFF cone bipolar cells due to the specificity of their wiring in the IPL. Most, but not all, ON cone bipolar cells can be labeled in this way but there is evidence that at least one ON cone bipolar type is not coupled to the AII network (see discussion; Petrides and Trexler, 2008; Sigulinsky et al., 2020). Bearing in mind this limitation, here we report that the dendrites of AII-coupled ON cone bipolar cells made contacts exclusively with cone pedicles. In agreement with the ultrastructural analysis of all ON cone bipolar inputs in the mouse retina (Behrens et al., 2016), our results do not support the presence of rod contacts with ON cone bipolar cells. In contrast to OFF cone bipolar cells where the direct rod input forms a third rod pathway, we did not find evidence for a tertiary ON pathway in the rabbit retina.

## Materials and Methods

### Preparation of Isolated Retina

All procedures conducted were approved by the Institutional Animal Welfare Committee. Adult New Zealand albino rabbits of either sex (2–3 kg) were used for this study. Rabbits were deeply anesthetized with urethane (loading dose, 1.5 g/kg, i.p.). Immediately prior to enucleation 2% lidocaine hydrochloride drops were applied topically to each eye. Retinas were isolated from the eyecup while immersed in carboxygenated Ames medium and mounted on 0.8 μm black filter paper. Retinal cells were pre-labeled with 4,6-diamino-2-pheynylindole (DAPI) by incubating retinal pieces in Ames medium containing 5 μM DAPI for 15 min.

### Neurobiotin Injection

Retinal pieces pre-labeled with DAPI were visualized on an Olympus BX-50WI microscope (Tokyo, Japan) equipped with epifluorescence and a 40× water immersion objective. The retina was mounted in a prefusion chamber (RC-22, Warner Instruments, Holliston, MA, USA) at 35°C. RBCs were faintly labeled with DAPI, deep in the inner nuclear layer, while AII amacrine cells were brightly labeled at the margin of the IPL. Targeted RBCs or AII amacrine cells were impaled under visual control with 150–200 MΩ glass electrodes (Warner Instruments, Holliston, MA, USA) pulled on a horizontal electrode puller (Sutter Instrument, Novato, CA, USA). Electrodes tips were filled with 4% Neurobiotin (Vector Laboratories, Burlingame, CA, USA) and 0.5% Lucifer Yellow-NH_4_ (Molecular Probes, Eugene, OR, USA) in 0.1 M phosphate buffer, then backfilled with 3 M LiCl. Impaled cells were injected with a biphasic current (± 1.0 nA, 3 Hz) for 4–5 min. Following the last injection, retinal pieces were fixed in 4% paraformaldehyde diluted in 0.1 M PBS for 30 min prior to further immunohistochemical procedures.

### Immunocytochemistry

Following fixation, retinal pieces were washed six times in 0.1 M phosphate buffer saline (PBS, pH 7.4) then blocked overnight in phosphate-buffered saline (PBS) containing 0.3% Triton-X 100 (TX-PBS) and 3% normal donkey serum (NDS) at 4°C. After the block, tissues were incubated for 5–7 days in primary antibodies (listed in Table 1) diluted in TX-PBS and 1% NDS at 4°C. Retinas were then washed 6 × 10 min followed by incubation overnight in species-specific donkey-raised secondary antibodies diluted in TX-PBS and 1% NDS at 4°C. Secondary antibodies were conjugated to the fluorescent markers Alexa-488, Cy3, and/or Alexa-647, dilution 1:200 (Jackson ImmunoResearch Laboratories, West Grove, PA, USA). Neurobiotin injected cells were visualized with streptavidin conjugated to Alexa-488 or Cy3, dilution 1:200 (Jackson ImmunoResearch Laboratories, West Grove, PA, USA). Following final washes, retinas were mounted in Vectashield (Vector Laboratories) on glass slides and cover-slipped for examination.

**Table 1:** A list of primary antibodies, source and dilution used in this project.

Antigen	Immunogen	Manufacturer, species type, catalog number	Dilution
mGluR6	Rabbit C-terminus (KTTSTVAAPPKGADTEDPK)	Massey lab, rabbit polyclonal	1:1,000
PKCα	Rat CT variable region	Sigma, St Louis, MO, USA (P 4334), rabbit polyclonal	1:500
PKCα	Amino acids 270–427 of human PKCα	BD Biosciences, San Jose, CA, USA, No. 610107, mouse monoclonal	1:500
Ribeye	CtBP2 C-term. a.a. 361–445	BD Biosciences #612044, mouse monoclonal	1:1,000
GluR5	C-terminus of human GluR5 (KLIREERGIRKQSSVHTV)	Santa Cruz Biotechnology, SC-7616, goat polyclonal	1:500
Calretinin	N-terminal peptide STVHEILCKLSLEGD	Millipore Sigma, SAB2500188	1:5,000
Calbindin	Recombinant rat calbindin D-28k	SWANT, Switzerland, CB38	1:1,000
Choline acetyl-transferase	Human placenta enzyme	Millipore Sigma, AB144P	1:500
vGlut1	Recombinant rat VGLUT1 (amino acids 456–500)	Synaptic Systems, Goettingen, Germany 135304	1:3,000

### Image Acquisition and Analysis

Confocal images were acquired on a Zeiss LSM-510 or a Zeiss LSM-780 (Zeiss, Thornwood, NY, USA) confocal microscope using a 63× (numerical aperture 1.4) oil immersion objective. Images were acquired at 1,024 × 1,024 pixels, 16 bits with 4× averaging. The gain and laser intensity were carefully adjusted for each channel to avoid saturation and reduce background noise. RBCs were difficult to image because of the large intensity range, from very bright cell bodies to relatively low-intensity dendrites. In this case, we set the gain to view the dendrites, resulting in saturation of the cell bodies. In some cases, this caused a minor artifact, visible as some horizontal banding (Figure 4), but it did not affect the data on connectivity. Three channel images were scanned sequentially in a series of 0.3–0.5 μm optical sections and ministacks of these images were constructed from 2–6 optical sections, to include structures of interest and flatten the tissue. The images presented here were obtained from wholemount preparations mounted ganglion cell side up/photoreceptor side down. The brightness and contrast of the images were adjusted using Adobe Photoshop v7.0 (Adobe Systems, San Jose, CA, USA). Sometimes images were exported to Imaris (Bitplane, Zurich, Switzerland) for Z-axis projections (Figures 7–9). As required, these projections were edited with the Imaris tools Slicer or Oblique Slicer, to reduce the projection depth and focus on structures of interest. No other filtering, editing, or enhancement was applied to any of the presented images.

**Figure 1:**
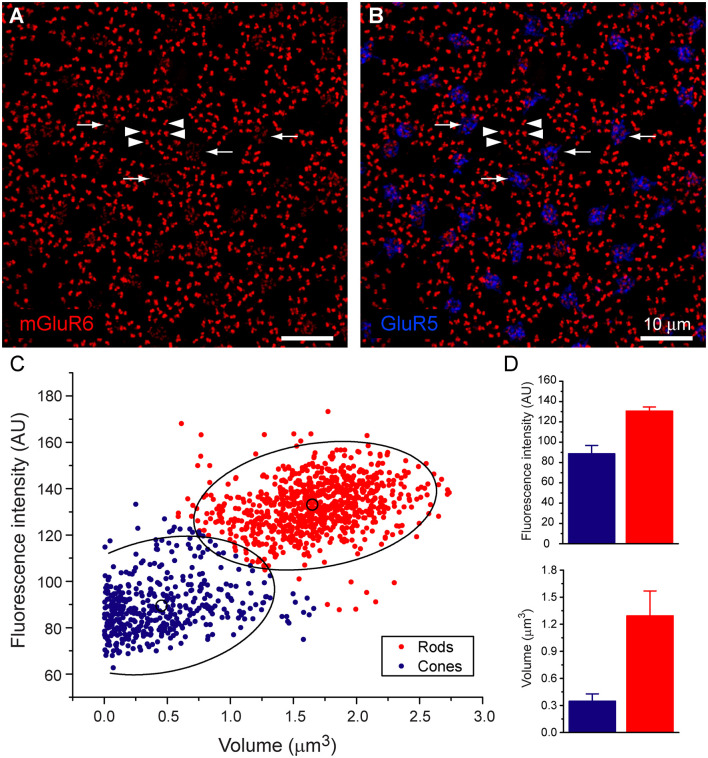
Rod and cone mosaic. **(A)** mGluR6 labeled puncta within the OPL of the rabbit retina reveal two separate labeling patterns. Large doublets, arrowheads, which correspond to the tips of two rod bipolar dendrites at each rod spherule and smaller clustered puncta which correspond to cone pedicles (arrow). **(B)** Addition of GluR5 labeling reveal the locations of cones. **(C)** Cluster analysis of mGluR6 puncta reveal that two distinct populations are readily statistically separable, ellipses show 95% confidence limits. **(D)** Mean volume and mean intensity of mGluR6 labeling (arbitrary units), (mean + SD, *n* = 3, *p* < 0.05).

**Figure 2:**
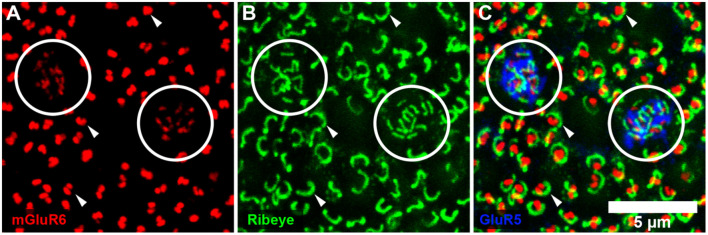
Rod and cone mosaic. **(A)** mGluR6 labeling in the OPL shows clusters of fine terminals, associated with cone pedicles (circled) and larger, brighter doublets, associated with rod spherules. **(B)** Same field labeled with an antibody against ribeye to stain synaptic ribbons shows a cluster of small ribbons associated with cone pedicles (circled) and larger horseshoe-shaped ribbons at rod spherules. **(C)** Triple label, same field, shows GluR5 labeling at cone pedicles (circled) with the smaller ribbons and mGluR6 clusters. At rod spherules, the curved synaptic ribbon encloses the mGluR6 doublets.

**Figure 3:**
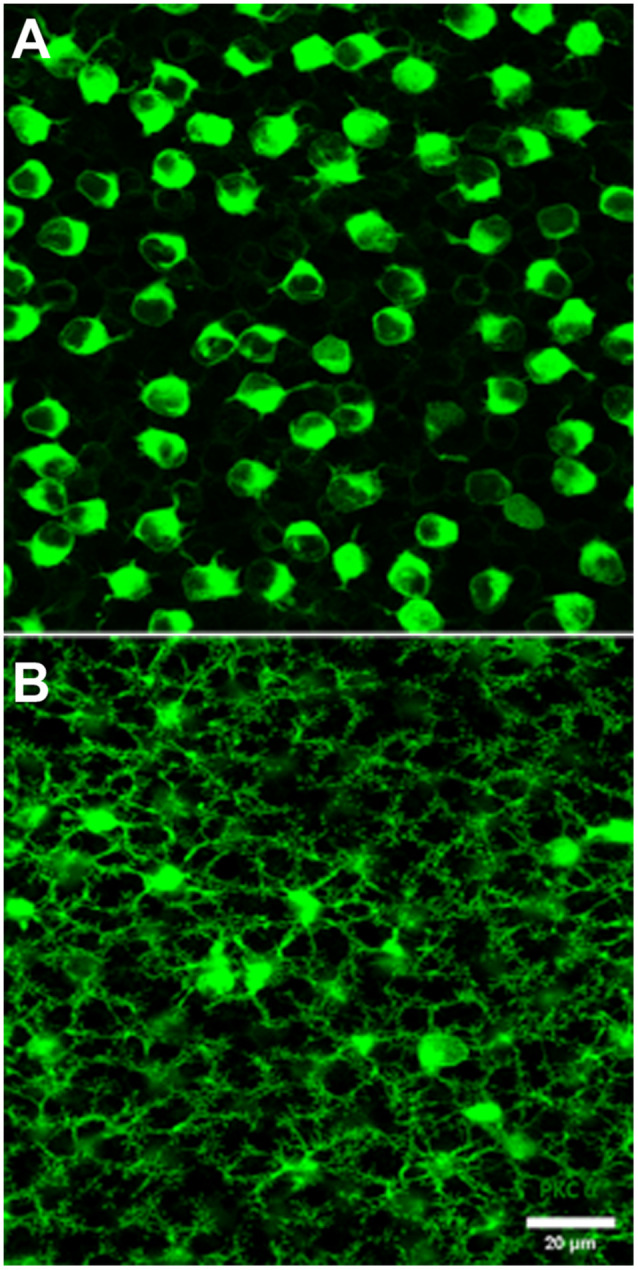
PKC labeling, rabbit retina, wholemount. **(A)** The cell bodies of rod bipolar cells are stained for PKC. **(B)** Focus in the OPL shows a large population of very fine processes which terminate at rod spherules.

**Figure 4:**
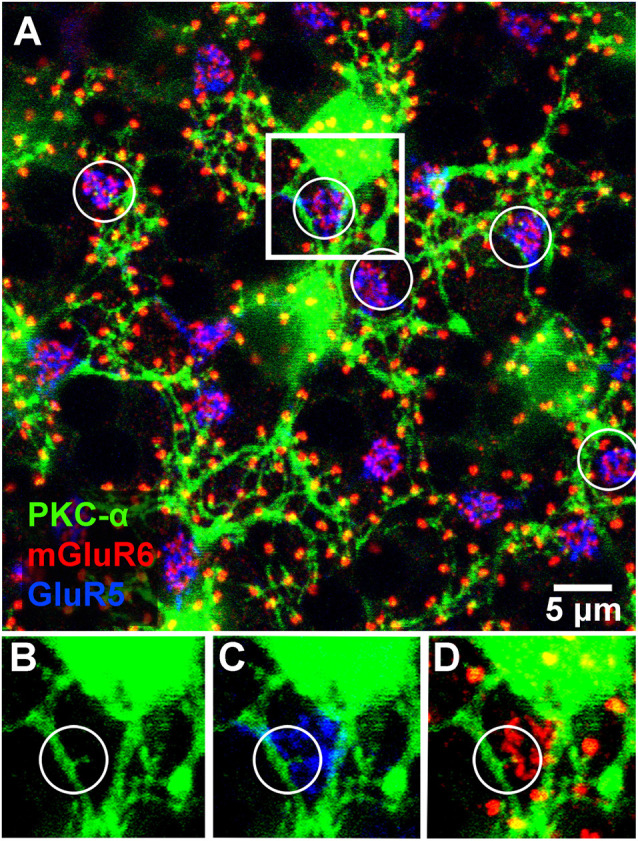
Triple label showing rod and occasional cone contacts of PKC labeled rod bipolar cells. RBCs receive putative contact from cones. **(A)** Labeling of rod bipolar cell dendrites at the level of the OPL with an antibody against PKC-α demonstrates that many mGluR6 clusters at rod spherules are double-labeled. Cone pedicles are shown by the combination of fine mGluR6 terminals and GluR5 labeling. Some cone pedicles, circled, receive dendritic contacts from PKC labeled rod bipolar cells. **(B–D)** enlarged view of the square area in **(A)** to show individual channels. **(B)** A very fine branch from a rod bipolar dendrite reaches out to a cone pedicle. **(C)** The rod bipolar dendrite contacts the cone pedicle labeled for GluR5. **(D)** The rod bipolar dendrite is double-labeled for mGluR6 where it terminates within the cone pedicle. Note: some horizontal banding in this image resulted from saturation of the RBC cell bodies, which were saturated because we set the gain to view the fine dendrites; it did not affect the analysis of connectivity.

**Figure 5:**
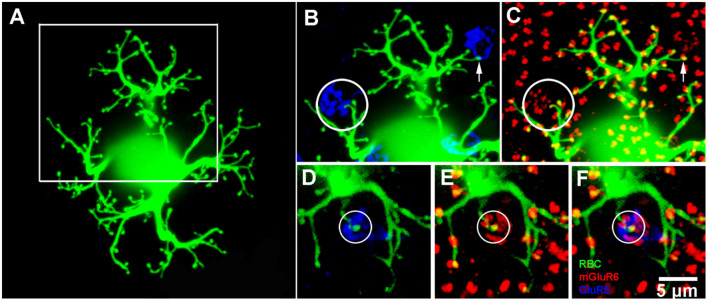
Rod bipolar cell—dye fills. **(A)** A single rod bipolar cell filled with Neurobiotin (green), focus on the dendrites in the OPL. The cell body is blurred because it is out of focus. This is a perfect fill which provides complete details of the dendritic tree. The major dendritic branches terminate at approximately 100 rod spherules. **(B)** Enlarged view of square in **(A)** to show two cone pedicles labeled for GluR5 (blue). The left cone pedicle, circled, receives a branch from the dye-filled rod bipolar cell. **(C)** Enlarged view of square in **(A)** to show the rod bipolar cell contact with a cone terminates at a fine mGluR6 (red) puncta within the cone pedicle, circled. On the right side, the rod bipolar cell dendrites appear to approach another cone pedicle but terminate at two close-by rod spherules. This can be seen because the rod bipolar dendrites terminate at one half of an mGluR6 doublet, arrow. This pattern is seen throughout the dendritic tree because normally rod spherules receive input from two different rod bipolar cells. **(D–F)** An example from another Neurobiotin filled rod bipolar cell where a rod bipolar dendrite reaches to the center of a cone pedicle, circled. Again, except for the cone contact, the rod bipolar dendrites terminate at half an mGluR6 doublet marking the site of rod spherules.

**Figure 6:**
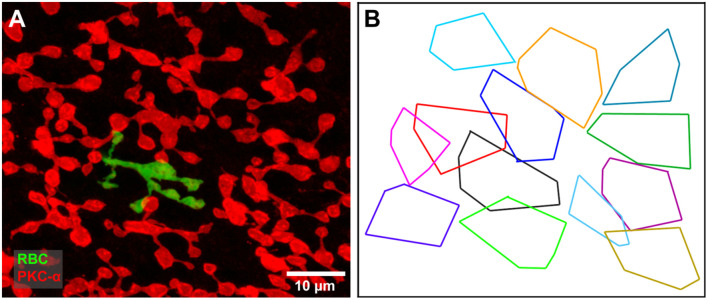
**(A)** A single dye filled rod bipolar cell (green) with cone contacts against a background of PKC labeled rod bipolar cells (red). Focus on the rod bipolar terminal deep in the IPL. **(B)** The axon terminal field of the filled rod bipolar cell (black) against a background showing the terminal fields of all PKC-labeled rod bipolar cells. The filled cell appears to be part of a single mosaic with a coverage of 0.75, typical of bipolar cells.

**Figure 7:**
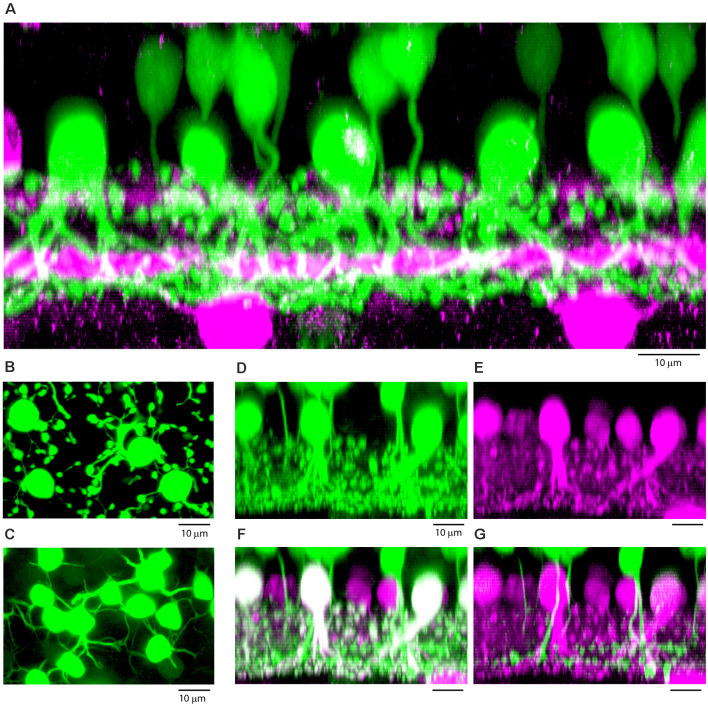
**(A)** Vertical section through a patch of dye-filled AII amacrine cells (green), which have stout descending dendrites and tethered lobules in sublamina a, and a dense plexus in sublamina b, beneath the lower cholinergic band (magenta), used as a depth marker. Above the AII amacrine cells, there are numerous tracer-coupled cone bipolar cells (also green), with smaller somas and axons which descend and become lost in the AII plexus. Z-axis projection, thickness, 18 μm. **(B)** Wholemount view of dye-filled AII amacrine cells showing typical morphology with tethered lobules, projection, thickness 10 μm. **(C)** Same field, focus in the OPL showing tracer-coupled ON cone bipolar cells with dendrites, projection, thickness 10 μm. **(D–G)** Subtractive labeling. **(D)** Section through a dye-filled AII patch, with overlying dye-coupled ON cone bipolar cells. **(E)** Same field, calretinin labeled AII amacrine cells (magenta). **(F)** Same field, double label showing dye-filled AII amacrine cells (Neurobiotin, green + calretinin, magenta = white). In the background there are other AII amacrine cells outside the dye-coupled patch, which are stained only for calretinin (magenta). Above the AII amacrine cells, there are dye-coupled ON cone bipolar cells (Neurobiotin only, green). **(G)** Subtracting the AII amacrine cells (magenta) isolates the cone bipolar cells (green) and reveals bipolar cells axons which descend to sublamina b of the IPL. Terminals at different depths show the presence of several different ON cone bipolar types. **(D–G)** Z-axis projection, thickness 15 μm.

**Figure 8:**
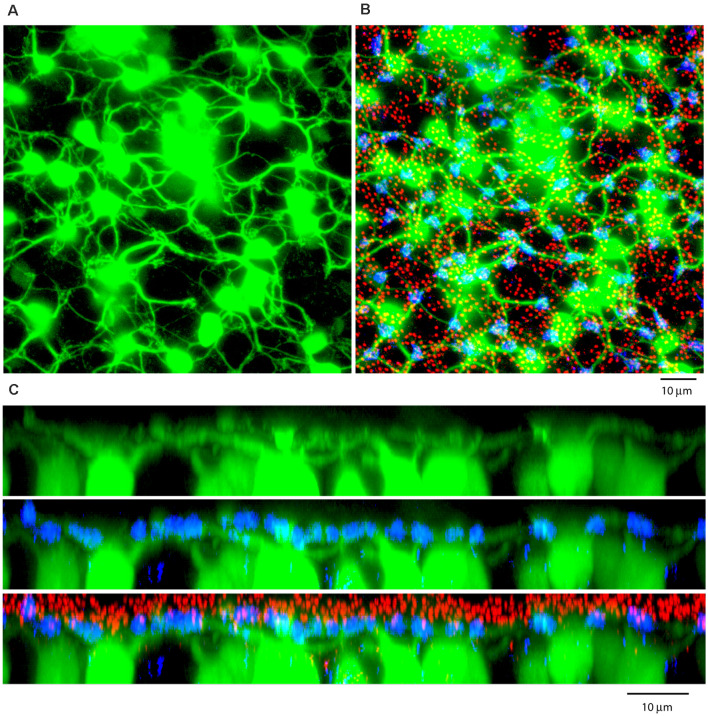
**(A)** A Neurobiotin-filled patch, following the dye injection of a single AII amacrine cell, shows a large number of dye-coupled ON cone bipolar cells (green) with dendrites in the OPL, projection, thickness 12 μm. **(B)** Labeling the rod-cone mosaic for mGluR6 (red: rod bipolar tips at rod spherules) and GluR5 (blue, cone pedicles) shows that the dye-coupled ON cone bipolar dendrites only contact cones, projection, thickness 12 μm. **(C)** Z-axis rotation to show a section through the same dye-coupled patch shows that most rod spherules lie substantially above both the cone pedicles and the dendrites of ON cone bipolar cells, projection, thickness 22 μm.

**Figure 9:**
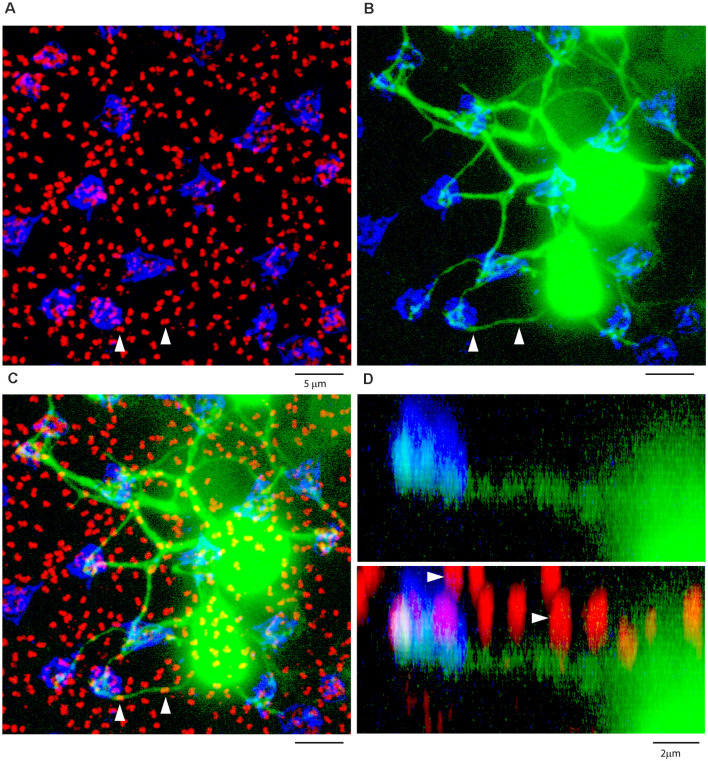
ON cone bipolar cells contact cones exclusively. **(A)** The rod-cone mosaic labeled for mGluR6 (rod spherules, red) and GluR5 (cone pedicles , blue), projection, thickness 8 μm. **(B)** Same field showing detail of dye-coupled ON cone bipolar cells (green) with dendrites reaching out to GluR5 labeled cone pedicles (blue), projection, thickness 8 μm. **(C)** There is no indication that ON cone bipolar cell dendrites terminate at mGluR6 labeled rod [spherules (red), projection, thickness 8 μm. A green dendrite passes underneath two rod spherules (white arrowheads). **(D)** Z-axis rotation shows that the bipolar dendrite is at a different depth than the two rod spherules (white arrows) and simply passes underneath without contact, projection, sliced in Imaris, thickness 2 μm.

### Subtractive Labeling

In multi-channel images, the general principle is that channels can be subtracted, as opposed to the more usual addition. Neurobiotin injections of AII amacrine cells stained the dye coupled-ON cone bipolar cells but their axons were rapidly lost as they descended into the dense meshwork of AII processes in the IPL. To circumvent this problem, we selectively labeled the AII population with an antibody against the calcium-binding protein calretinin, which is specific for rabbit AII amacrine cells at a high dilution (1:5,000; Massey and Mills, 1999). Arithmetic calculations were performed in Zen (Zeiss, Oberkochen, Germany) using Image Calculator. The AII-specific calretinin signal was doubled and then subtracted from the Neurobiotin signal. This has the effect of isolating the Neurobiotin-only bipolar cells for clarity.

### Quantification of mGluR6 Puncta

Confocal images of flat mount retina, labeled with mGluR6, were obtained ensuring the brightest plaques were not saturated. Measurements of volume and intensity for mGluR6 labeling in the OPL were made using Imaris software and the surface tool. A ministack of 10–20, 0.4 μm optical sections was acquired at the focal plane of the OPL to include all mGluR6 puncta. A total of three retinae, each including 40–50 cones and 700–800 rods, were analyzed for statistical analysis.

### Statistics

For mGluR6 analysis, a principal components analysis was used to generate two clusters (K means, R4.0.2 package “cluster” (Vienna, Austria). The larger, brighter points were identified as rod mGluR6 receptors and the points were color-coded. Ellipses were drawn to show 95% confidence limits. Plots to show mean intensity (arbitrary units, AU), and size with standard deviation were produced in Origin (Northampton, MA, USA).

## Results

### Photoreceptor Mosaic

To determine whether RBCs receive direct input from cone photoreceptors, we first needed to establish how to visualize these connections. An antibody against mGluR6 labels the post-synaptic glutamate receptor of all depolarizing bipolar cells. Thus, mGluR6 labeling is located on the dendritic tips of both ON cone bipolar cell and rod bipolar cells (Nomura et al., 1994; Vardi and Morigiwa, 1997; Li et al., 2004). The labeling pattern of mGluR6 reveals the location of the rod and cone mosaic (Figure 1A). This labeling pattern has been used previously to determine rod input to OFF cone bipolar cells (Li et al., 2004).

At first glance, two types of mGluR6 puncta arise. One type comes in the form of large, bright doublets, which indicate the tips of rod bipolar dendrites, usually two per rod spherule. The other type consists of a cluster of smaller, low-intensity puncta which mark the dendritic tips of ON cone bipolar cells and are thus associated with cones (Figures 1A,B). To confirm this, we measured the intensity and volume of mGluR6 puncta and performed a cluster analysis. Our results provide clear evidence of two independent groups of mGluR6 when clustered by intensity and volume (Figures 1C,D). While volume provides the most significant visual aid in differentiating the rods from cones, there was also a significant difference in intensity. As expected, rod associated mGluR6 puncta were significantly more intensely labeled, 1,307 + 40 AU (mean + SD, *n* = 3, *p* = 0.03) than cone associated mGluR6 puncta, 885 + 83 AU (mean + SD, *n* = 3, *p* < 0.01). Additionally, rod mGluR6 puncta were significantly larger, 1.29 ± 0.28 μm^3^ (mean + SD, *n* = 3) than cone mGluR6 puncta, 0.35 + 0.08 μm^3^ (mean + SD, *n* = 3). These data provide evidence that there are significant differences between rod and cone mGluR6 puncta.

To further confirm our mapping of the rod and cone mosaic, we double-labeled with an antibody to GluR5. GluR5 precisely labels cone terminals within the outer plexiform layer across species (Haverkamp et al., 2001, 2003). Figure 1B shows that clusters of mGluR6 labeled cone bipolar terminals are associated with GluR5 labeled cone pedicles. We can also visualize the location of the rod and cone mosaic using an antibody against ribeye, which labels the synaptic ribbon found within both rods and cones (Schmitz et al., 2000; Tom Dieck et al., 2005). Figure 2 clearly shows two different populations of ribeye labeling. One population labels a horseshoe-shaped single ribbon that is associated with rod spherules and the other population of smaller and straighter ribbons that are associated with cones. Together these data demonstrate our ability to accurately differentiate between rod input vs. cone inputs.

### Rod Bipolar Cell—Photoreceptor Contacts

Previous EM analysis from HRP filled RBCs demonstrated that one out of two received input directly from cones (Dacheux and Raviola, 1986). The sample size for this work, however, was very limiting. To avoid sampling errors and demonstrate the possibility of cone input to RBCs, we labeled the entire population of RBCs with an antibody against PKC-α (Greferath et al., 1990). PKC-α labels not only the entire population of RBCs (Figure 3A), but it also labels their dendrites (Figure 3B) found within the outer plexiform layer. Using this antibody in combination with mGluR6 and GluR5, we were able to identify the photoreceptor input to all RBCs (Figure 4). The vast majority of RBC dendritic tips, approximately 100 per RBC in rabbit retina (Pan and Massey, 2007), ended at and colocalized with rod associated mGluR6 puncta. Closer inspection revealed that some RBC dendrites end, not at a rod spherule, but instead terminated within the boundary of a cone pedicle. Furthermore, the terminal dendrite colocalized with a cone associated mGluR6 puncta. Unfortunately, with this technique, it was difficult to adequately quantify the number and quality of the contacts. The problem is that rod bipolar dendrites are very fine, and they may have relatively low levels of PKC, certainly compared to the soma. However, these data do provide evidence for direct contacts between cones and RBCs.

To determine, unequivocally, whether a subset of RBCs receive direct cone input in the rabbit retina, we dye-injected RBCs with Neurobiotin. RBCs were visually targeted with DAPI and following streptavidin labeling were processed with mGluR6 and GluR5 antibodies. Dye filled cells were determined to be RBCs based on the characteristic morphology of their dendrites (Figure 5A), typically short stubby branches with bulbous tips, and by the stratification of their large axon terminals at the proximal end of the inner plexiform layer (data not shown; Strettoi et al., 1990; Young and Vaney, 1991). Some dye-filled cells were co-labeled with PKC-α to further confirm their identity (data not shown). The majority of dendritic terminals (~100) were colocalized with the large mGluR6 doublets characteristic of rods. Furthermore, only one of each doublet was double-labeled because the two RBC dendrites which invaginate each rod spherule typically come from different cells (Figure 5A). In a subset of injected RBCs, however, 1–2 terminal branches ended not only within the boundary of a cone pedicle, but also colocalized with a cone associated mGlur6 punctum (Figures 5B–F). In the enlarged view of Figures 5B,C, a rod bipolar dendrite reaches out to a cone pedicle (circled) where it is colocalized with a small mGluR6 cluster typical of cones. Another example is shown in Figures 5D–F, where an RBC dendrite penetrates to the center of a cone pedicle. These are cone contacts with an RBC. In other cases, RBC dendrites sometimes approached a cone pedicle, but as seen by the colocalization with one part of an mGluR6 doublet, these were rod spherule contacts merely adjacent to a cone pedicle and were not counted (arrow, Figures 5B,C). In total, we found that 16 of 28 (~57%) Neurobiotin injected RBCs received at least one, but sometimes two, cone inputs.

Pang et al. (2010) suggested that RBCs are split into two functional subsets based on their photoreceptor inputs. To test this hypothesis, we examined the axonal tree of a dye-injected RBC that received cone inputs. If the cone-connected RBCs are a different cell type, they should not form part of the regular mosaic of RBC axonal terminals labeled with PKC-α. However, the dye-injected, cone-connected RBCs fitted well with the tiling of all RBCs (Figure 6). We found that the axon terminals of RBCs provide a coverage factor of 0.75 which is consistent with RBCs found within the superior rabbit retina (Young and Vaney, 1991) and typical of bipolar cells in general. Based on this coverage factor, it is very likely that the tiling pattern of RBCs represents a single cell type, not two. Similar results suggest that mouse RBCs belong to one population, even though they exhibit variability in both physiology and cone connections (Tsukamoto and Omi, 2017; Pang et al., 2018).

### AII Amacrine Cell Fills

For technical reasons, including the targeting and/or identification of specific cone bipolar cell types, we did not think it was practical to address the photoreceptor connectivity of cone bipolar cells by individually filling cone bipolar cells. Therefore, we chose to adopt an alternative method, by filling AII amacrine cells. Because AII amacrine cells are coupled to most ON cone bipolar cells, this yields a bulk population of ON cone bipolar cells filled with Neurobiotin *via* the gap junctions with AII amacrine cells in the IPL, as first documented by David Vaney (Vaney, 1991), reflecting the gap junction connections reported in early anatomical studies (Famiglietti and Kolb, 1975).

An example of a single AII injection is shown in Figure 7. In a projection, a number of prominent dye-coupled AIIs, identified by their morphological characteristics, are located in the INL, adjacent to the IPL (Figure 7A). Each well-labeled AII had a stout primary dendrite descending to the bottom of the IPL. In addition, there were numerous lobules tethered by fine dendrites in sublamina a. These are the well-recognized properties of AII amacrine cells. In addition, a large number of bipolar cells, with somas relatively high in the ONL, were also filled, *via* their gap junction connections with AIIs. The magenta labeling shows the ChAT bands as a stratification reference. The bipolar cell axons descend into sublamina b, sometimes below the lower cholinergic band, and they are thus identified as ON cone bipolar cells. However, intermingling with the AII dendrites precludes the identification of individual bipolar cells. In the wholemount view, a mosaic of AII somas was surrounded by many lobules in sublamina a. In the same field, the overlying dye-coupled ON cone bipolar cells produce a matrix of fine dendrites in the OPL. In this subfield, more than a dozen ON cone bipolar cells were well-labeled. The mosaic properties of bipolar cells, by which adjacent cells are likely to belong to different classes, immediately suggests that many types of ON cone bipolar cells are labeled. In summary, we conclude that many ON cone bipolar cells, of several distinct classes are labeled by the transfer of Neurobiotin through gap junctions with AII amacrine cells.

### Subtractive Immunolabeling

Following AII injections, the details of bipolar axon terminals were obscured by the dendrites of the AIIs. In a side projection, the AII dendrites and the bipolar cell terminals were intermingled (Figure 7D). To overcome this problem, we labeled the AII amacrine cells with an antibody against the calcium-binding protein calretinin (Massey and Mills, 1999; Figure 7E). When the two channels were merged, the combination showed the dye-coupled AII amacrine cells in white (green plus magenta) with the more distant AIIs in the background with low Neurobiotin levels stained only for calretinin (magenta; Figure 7F). Masking and subtracting reveals the isolated ON cone bipolar cells with axons that clearly descend to sublamina b (Figure 7G). Furthermore, the axons terminate in several different strata, on both sides of the lower cholinergic band, indicating that several different bipolar cells can be labeled and distinguished by this procedure. We made no attempt to classify the rabbit ON cone bipolar cells, but the deepest stratification identifies some of the dye-coupled cells as calbindin bipolar cells. This was confirmed in some dye-injected patches where Neurobiotin labeled bipolar cells could be double-labeled for calbindin.

### On Cone Bipolar Cell—Photoreceptor Contacts

We used the same strategy to label the photoreceptor mosaic as previously described. A large patch of dye-coupled ON cone bipolar cells produced a matrix of overlapping dendrites in the OPL (Figure 8). Often, it could be observed that dendrites from several different cells converged at the same spot. When superimposed on a map of the photoreceptor terminals, these common sites were readily identified as cone pedicles by the combination of mGluR6 and GluR5 labeling. In a projection of the same material, the bipolar dendrites converged to cone pedicles labeled with GluR5 but the vast majority of rod spherules, marked by mGluR6, were above the cone pedicles, beyond the reach of the ON cone bipolar dendrites (Figure 8).

In a high-resolution view of several ON cone bipolar cells focused in the OPL, their dendrites converged at the GluR5 labeled cone pedicles (Figure 9). As they traverse the OPL, the bipolar dendrites are smooth with no branches or twigs but when they reach a cone terminal, there are often several branches and terminal swellings which are colocalized with mGluR6 receptors, as expected for an ON cone bipolar cell. These terminal specializations indicate synaptic input. In contrast, when the bipolar dendrites cross an mGluR6 labeled rod spherule by chance, there are no branches nor any morphological sign of swelling or terminal that would suggest a synaptic contact. In fact, the ON cone bipolar cell dendrites do not contact the rod spherules; they are merely passing by. This is readily apparent in a Z-axis projection, where two rod spherules, marked by arrowheads, that apparently overlap a rod mGluR6 doublet, were clearly separated in-depth (Figure 9). In other words, the bipolar dendrites pass under the rod spherules, en route to a cone pedicle. From four different dye-injected patches, containing a total of approximately 160 ON-cone bipolar cells, we were unable to find any rod contacts. There were many examples of apparent overlap in the XY view between ON cone bipolar dendrites and the large mGluR6 doublets characteristic of rod spherules. But careful examination in XZ or YZ projections of 209 examples showed they were always separated by depth and never colocalized. In summary, it was a simple matter to show that the dendrites of many ON cone bipolar cells converged at points readily identified as cone pedicles. In contrast, we were unable to demonstrate any synaptic contacts or synaptic specializations between ON cone bipolar dendrites and rod spherules.

### Blue Cone Bipolar Cells

**Figure 10:**
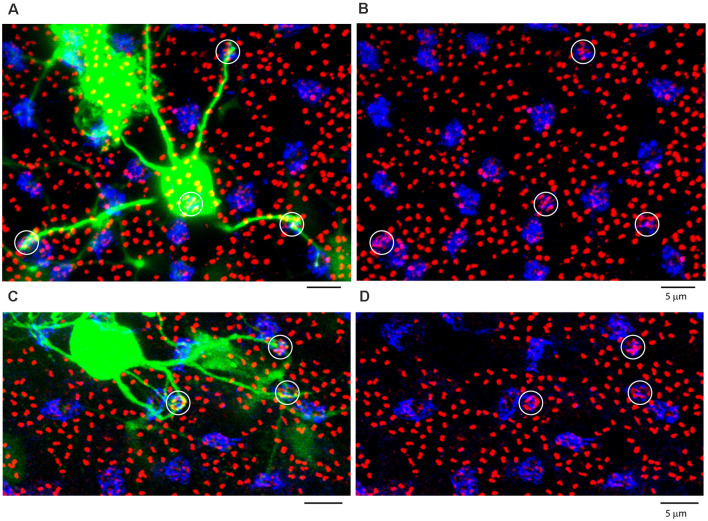
Blue cone bipolar cells contact blue cones exclusively. **(A,C)** Two separate blue cone bipolar cells, both dye coupled to an injected AII patch. They are identified as blue cone bipolar cells by their long eccentric dendrites which bypass other cones to terminate at blue cones. **(B,D)** Same fields, with blue cones circled, with weak GluR5 labeling and prominent mGluR6 terminals. All panels, projection, thickness 10 μm.

The total number of dye-filled ON cone bipolar cells was sufficient to include examples of all dye-coupled types. This was confirmed by identifying a few blue cone bipolar cells, which are the least numerous of all bipolar cell types (Behrens et al., 2016; Sigulinsky et al., 2020). The diagnostic criteria were long frequently asymmetric dendrites, which often bypassed other cones to make contact exclusively with blue cone pedicles (Kouyama and Marshak, 1992; Figure 10). Sometimes, a single dendrite from a blue cone bipolar cell would form a terminal hook which could be colocalized with most of the mGluR6 clusters at a single blue cone pedicle. Blue cone pedicles could be reliably identified by the weak labeling for GluR5 and the presence of a few prominent mGluR6 clusters. In addition, blue cone pedicles were often adjacent to other cones and did not obey the mosaic/nearest neighbor rules. As shown in Figure 10, more than one dendrite from the same blue cone bipolar cell could converge on the same blue cone. Following the axons of blue cone bipolar cells showed that they stratified deep in sublamina b of the IPL, below ChAT b. As with other types of ON cone bipolar cells, blue cone bipolar cells made no branches or terminals on their way to contact a specific cone. We could find no evidence that blue cone bipolar cells had any contacts with rod spherules.

## Discussion

Using individual dye-filled rod bipolar cells and a population of ON cone bipolar cells selectively labeled *via* gap junctions with AII amacrine cells, we have examined the connectivity between photoreceptors and bipolar cells in the rabbit retina. We found occasional cone contacts with approximately half the rod bipolar cells, confirming previous work in the mouse retina (Behrens et al., 2016; Pang et al., 2018). However, AII-coupled ON cone bipolar cells were exclusively in contact with cones. These results suggest a limited cone input to the primary rod pathway but, in the rabbit retina, we found no evidence for the ON equivalent to the tertiary rod pathway mediated by rod input to certain OFF cone bipolar cells.

### Rod Bipolar Cell Connections

Three separate pathways have been studied that convey scotopic signals across the retina. The primary rod pathway is the direct connection of rods to RBCs and then to AII amacrine cells, which route the signal to both ON and OFF retinal ganglion cells. The secondary rod pathway consists of electrical coupling between rods and cones *via* gap junctions and the tertiary rod pathway is carried by direct connections from rods to certain OFF cone bipolar cells. These latter two pathways are less studied but thought to merge rod signals into cone pathways in the mesopic range of light intensities.

In the current study, we have examined a potentially novel pathway that involves direct cone photoreceptor input to RBCs. We found, using a morphological analysis, that a subset (~57%) of all RBCs receive direct input from one or two cone photoreceptors. Thus, we have confirmed the original work of Dacheux and Raviola (1986), who reported that one of the first reconstructed RBCs received a cone contact in addition to numerous rod inputs. The cone input is relatively small because each rod bipolar cell in the rabbit retina receives approximately 100 rod contacts (Pan and Massey, 2007). There are also one or two cone inputs to half or more of the RBCs in the mouse retina and, conversely, more than half the cones contact RBCs (Behrens et al., 2016; Pang et al., 2018). Although these are sparse connections, the frequency, more than half of the RBCs receive cone contacts, suggests a repeated pattern rather than a developmental error or misconnection. Finally, the presence of cone input to rod bipolar cells in mouse, rabbit and primate retina (Behrens et al., 2016; Pang et al., 2018) suggests this may be a conserved feature of the mammalian retina.

The functional significance of cone input to a subset of RBCs is unclear. Pang et al. (2010) showed that receiving cone input extends the dynamic working range of RBCs. Multiple studies have demonstrated that RBCs saturate at light levels far below the cone photoreceptor threshold. A recent study, however, has shown that RBCs may not saturate at low light levels as previously thought, but instead operate over a much larger range (Ke et al., 2014). The functions of RBCs switch from sensitive photon detection to contrast detection. In addition to extending the dynamic range, this may play an important role in crossing over from scotopic to photopic vision.

Our data on the tiling of RBCs in the rabbit retina, with or without cone connections, does not support the presence of two separate types of rod bipolar cell, as proposed by Pang et al. (2010). A detailed analysis of mouse RBCs showed that although there was variation in RBC morphology, they all belonged to a single type (Tsukamoto and Omi, 2017; Pang et al., 2018). The serial blockface EM data for mouse found more than half of RBCs receive cone input but failed to discriminate between those with or without cone contacts on the basis of rod contacts, connectivity, stratification, or mosaic properties (Behrens et al., 2016). In addition, genetic analysis of mouse bipolar cells reported only one cluster for rod bipolar cells while separating the cone bipolar cells into 13 types, which correspond to known morphological types (Shekhar et al., 2016). Thus, the weight of available data suggests that RBCs in mammalian species make up only one cell population. Still, within this population, there is some variability in RBC morphology, physiological responses, and the presence or absence of direct cone contacts (Pang et al., 2010, 2018; Behrens et al., 2016 Tsukamoto and Omi, 2017).

### The Tertiary Rod Pathway: Connections Between Rods and Certain OFF Cone Bipolar Cells

There is strong evidence for the connection between rods and certain OFF cone bipolar cells, as first proposed by Soucy et al. (1998) and Li et al. (2004). Specifically, OFF bipolar types 3A, 3B, and 4 all make basal contacts with rod spherules in the mouse retina (Tsukamoto and Omi, 2014; Behrens et al., 2016). These contacts coincide with excitatory glutamate receptors of the AMPA/kainate subtype providing a sign-conserving input consistent with the physiological responses of OFF cone bipolar cells (Hack et al., 1999). In addition, the direct connection between rods and certain OFF cone bipolar cells was demonstrated by paired recording in the ground squirrel retina (Li et al., 2010). Direct rod input to OFF cone bipolar cells constitutes the tertiary rod pathway in the mammalian retina. Although the anatomical physiological basis for the tertiary rod OFF pathway is convincing, the function of this pathway is largely unknown, although it is thought to operate in the high scotopic/mesopic range.

### Absence of ON Cone Bipolar Cell Contacts With Rods

The slow scotopic input to cone bipolar cells reported by Pang et al. (2010) suggests the presence of direct connections between rods and ON cone bipolar cells, mediated *via* sign-inverting mGluR6 receptors. Contacts between rods and ON cone bipolar dendrites have been reported in mice, specifically for type 7 bipolar cells (Tsukamoto et al., 2007; Keeley and Reese, 2010; but see Haverkamp et al., 2006) and for giant ON bipolar cells in primate retina (Tsukamoto and Omi, 2016). The alignment of bipolar cell types across species is problematic, thus the rabbit equivalent of the mouse type 7 is unclear. In the mouse, eight ON cone bipolar cell types have been found, based on the analysis of thousands of bipolar cells (Shekhar et al., 2016) but only seven types have been identified in the rabbit, based on a smaller dataset (Sigulinsky et al., 2020). Sigulinsky et al. (2020) suggest two types comparable to the mouse type 7, rabbit bipolar cells CBb5 and CBb6, both of which make gap junctions with AII amacrine cells, like the mouse type 7, so they should be included in the group of cone bipolar cells filled *via* AII dye injections.

Here we report that AII-coupled ON cone bipolar cells in the rabbit contact cones exclusively and we could not identify rod contacts. In general, cone bipolar dendrites terminate low in the OPL, at the level of cone pedicles (Figure 8; Keeley and Reese, 2010). Thus, they cannot reach most rod spherules, which are found in several rows above the cone pedicles. More specifically, cone bipolar dendrites did not terminate at rod spherules and when they passed close to rod spherules, there were no branches, terminals, or dendritic specializations to suggest a synaptic connection. In the mouse retina, serial blockface EM reconstruction of all bipolar photoreceptor contacts revealed rod contacts with a subset of OFF cone bipolar cells. But, in agreement with the present work, there were no contacts between the rod and ON cone bipolar cells (Behrens et al., 2016).

We use the phrase AII-coupled ON cone bipolar cells intentionally because there is evidence that some cone bipolar cells do not make gap junctions with AII amacrine cells. In the mouse retina, Tsukamoto and Omi (2013) have suggested that one type does not receive AII input and therefore does not receive input from the primary rod pathway *via* RBC and AII. In the rabbit retina, a subset of ON cone bipolar cells contained no glycine (Petrides and Trexler, 2008). Since glycine is thought to diffuse from AII amacrine cells *via* gap junctions (Vaney et al., 1998), this implies that at least one ON cone bipolar cell is not coupled to AII amacrine cells and so would not be represented in the current experiments. This unexpected result was confirmed following the serial EM reconstruction of bipolar cell circuits in the rabbit retina (Sigulinsky et al., 2020). Most ON cone bipolar cells were coupled both to AII amacrine cells and within class to other ON cone bipolar cells. However, certain ON cone bipolar cells (CBb3 and CBb4) made few or no gap junctions with AII amacrine cells (Sigulinsky et al., 2020). Paradoxically, all ON cone bipolar cells had significant glycine levels, regardless of AII coupling. It was suggested that glycine may enter non-AII coupled ON cone bipolar cells *via* gap junctions with other ON bipolar types or other glycinergic amacrine cells (Sigulinsky et al., 2020). Thus, it is unclear whether all ON cone bipolar cells can be labeled *via* coupling to AII amacrine cells and we must concede the theoretical possibility that a non-AII coupled ON bipolar type could make rod contacts that would be overlooked by network labeling with Neurobiotin. Nevertheless, our results show that most ON bipolar cell types in the rabbit retina do not receive rod contacts, confirming an ultrastructural analysis of bipolar cell connectivity in the mouse retina which reported rod contacts only with certain OFF bipolar cell types (Behrens et al., 2016). Together, these results do not support the presence of a tertiary ON rod pathway in these two mammalian species.

In support of this conclusion, ganglion cell recordings from the mouse retina provide further evidence against a tertiary ON pathway (Deans et al., 2002). In the Cx36 knock-out mouse, there was a deficit in the rod ON responses; the absence of AII/ON bipolar cell gap junctions eliminated the primary rod pathway and the absence of rod/cone coupling eliminated the secondary rod pathway. This should then reveal the presence of a tertiary ON pathway with responses below the cone threshold. Yet the sensitivity of ON ganglion cell responses matched the cone threshold, suggesting the absence of a tertiary rod ON pathway.

In more recent work, Pan et al. (2016) suggested that inhibitory masking by GABA, mostly at the level of bipolar terminals, was responsible for selectively blocking different rod pathways to ganglion cells, thus producing a range of sensitivities. As before, in the Cx36 knock-out mouse, rod ON signals were diminished, and ON ganglion cell sensitivity was not enhanced by GABA antagonists. Presumably, this reflects the absence of primary and secondary rod pathways in the Cx36 knock-out mouse, and perhaps the lack of a tertiary ON pathway.

However, in multi-electrode array recordings, others have shown that while scotopic ON responses were reduced in the Cx36 knock-out mice, there were still some Cx36-independent ON responses at rod intensities (Cowan et al., 2016; Seilheimer et al., 2020). The ganglion cell types and the exact pathway for these responses are unknown. In the Cx36 knock-out, there should be no AII gap junctions and no rod/cone coupling, hence no primary or secondary rod pathways. Thus, a potential tertiary ON pathway was an attractive solution. Unfortunately, the most reliable morphological data, from serial EM reconstruction in mouse retina (Behrens et al., 2016), plus the data presented here from rabbit retina, do not support direct connections between rods and ON bipolar cells. Thus, there is some disagreement between the anatomy and the physiology that may require additional data to resolve.

In summary, while it is well-accepted that rod/OFF cone bipolar cell contacts support the tertiary rod OFF pathway, the weight of evidence suggests there are no direct contacts between rods and ON cone bipolar cells in rabbit and mouse. This implies that the contacts required for a tertiary rod ON pathway are not present across these two mammalian species (but see Tsukamoto et al., 2007 for the mouse b7 bipolar type and Tsukamoto and Omi, 2016 for giant ON bipolar contacts with rods). While it is always difficult to prove the absence of a connection or structure, the proposed absence of a tertiary ON pathway will be important for the design of experiments to investigate the contributions of the different rod pathways. It simplifies the situation for ON ganglion cells, which may only receive rod input *via* the primary and secondary rod pathways. To investigate the contribution of the tertiary rod pathway, it may be necessary to study OFF ganglion cells, specifically those which receive input from the OFF cone bipolar cells with direct rod input, as detailed by the analysis of connectivity in the mouse retina (Behrens et al., 2016).

### Blue Cone Bipolar Cells

Blue cone bipolar cells can be readily identified because of their selective contacts with blue cones. We found several clear and unambiguous examples among the AII-coupled ON cone bipolar cells. This provides unequivocal evidence that blue cone bipolar cells are coupled to AII amacrine cells, as previously reported based on overlapping dendrites and the presence of Cx36 immunolabeling (Field et al., 2009). Thus, the primary rod pathway includes blue cone bipolar cells and blue driven ganglion cells should receive scotopic input *via* the primary rod pathway. The secondary rod pathway is also viable because there is good evidence that blue cones make gap junction contacts with rods (O’Brien et al., 2012). However, there is no tertiary rod pathway to blue cone bipolar cells. In 4/4 examples, blue cone bipolar cells contacted blue cones exclusively and there was no evidence for contact with rods. This is consistent with the reconstruction of blue cone bipolar dendrites in the mouse retina which showed exclusive contacts with a subset of cones, thus identified as blue cones (Behrens et al., 2016). Blue cone bipolar cells form a subset of the ON cone bipolar cells and in common with the other cone bipolar cells make synaptic contacts only with cones. Thus, we found no anatomical evidence for a tertiary rod ON pathway in the rabbit retina.

### Asymmetry of ON and OFF Pathways

Across mammalian species, several ganglion cell types are present as paramorphic pairs, e.g., ON and OFF parasol and midget ganglion cells in primate, and alpha ganglion cells in many species. Thus, there may be some expectation that retinal pathways should be symmetrical. However, the physiological responses of paramorphic pairs are different, and there is clear asymmetry in their synaptic inputs (Zaghloul et al., 2003). In this article, we put forward the case that there is no contact between rods and ON cone bipolar cells even though direct contacts between rods and OFF cone bipolar cells are well established and accepted. Thus, there appears to be no ON equivalent of the tertiary OFF pathway, another ON/OFF asymmetry (but see Cowan et al., 2016 for Cx36-independent rod ON responses, and Tsukamoto et al., 2007 for potential rod contacts with type 7 bipolar cells in mouse retina).

What then could be the function of the asymmetrical tertiary OFF pathway? Only a subset of OFF bipolar cells receives direct input from rods, namely types 3a, 3b, and 4 in mouse retina (Behrens et al., 2016). These stratify in sublamina a of the IPL, just below the cholinergic a band, well-placed to contact OFF alpha ganglion cells, which have been associated with escape behavior in mice (Münch et al., 2009; Kim et al., 2020). A looming stimulus, such as a hawk, may present as a dark approaching object against a lighter background of sky, i.e., an OFF stimulus. Perhaps the tertiary OFF pathway provides extra input for this critical pathway. It is not as sensitive as the primary rod pathway but many raptors hunt at twilight. While it is interesting to speculate, further physiological experiments will be required to test such a hypothesis.

## Data Availability Statement

The datasets presented in this article are not readily available because they are image series. Requests for original confocal image data should be addressed to steve.massey@uth.tmc.edu.

## Ethics Statement

The animal study was reviewed and approved by UTHealth Animal Welfare Committee.

## Author Contributions

SCM, CW and GN designed the project. CW, GN and SCM carried out experiments. CW, GN, MI and SCM analyzed the data and produced figures. CW and SCM wrote the manuscript. All authors contibuted to the article and approved the submitted version.

## Conflict of Interest

The authors declare that the research was conducted in the absence of any commercial or financial relationships that could be construed as a potential conflict of interest.
